# Duck Tembusu Virus Exhibits Pathogenicity to Kunming Mice by Intracerebral Inoculation

**DOI:** 10.3389/fmicb.2016.00190

**Published:** 2016-02-19

**Authors:** Jinfeng Ti, Min Zhang, Zhijie Li, Xiuli Li, Youxiang Diao

**Affiliations:** ^1^Zoology Institute, Shan Dong Agricultural UniversityTai’an, China; ^2^Shandong Vocational Animal Science and Veterinary CollegeWeifang, China

**Keywords:** Tembusu virus, pathogenicity, Kunming mice, viral loads, animal model

## Abstract

In this study, Kunming mice were used as the animal models to study the pathogenicity of TMUV. Three groups of 3-week-old female Kunming mice (*n* = 15 mice per group) were infected with the SDSG strain of TMUV in 50 μL allantoic fluid (10^4.8^ ELD_50_/0.2 ml) respectively by the intracerebral (i.c.), subcutaneous (s.c.) and intranasal (i.n.) routes. The control group (*n* = 15 mice) was inoculated with 50 μL sterile phosphate-buffered saline. Clinical signs, gross, and microscopic lesions, viral loads in different tissues, and serum antibody titers were examined and recorded. Kunming mice infected intracerebrally showed typical clinical symptoms, including severe hindlimb paralysis, weight loss and death. Only dead mice presented severe intestinal mucosal edema. No gross lesions were observed in mice sequentially euthanized. However, microscopic lesions in the brain, spleen, liver, kidney, and lung were very typical including varying degrees of viral encephalitis, lymphocytes depletion, liver cell necrosis and nephritis, etc. Viral loads in different tissues were detected by the SYBR Green I real-time PCR assay. Viral loads in the brain, liver, and spleen were first detected and maintained a longer time, which indicated that these organs may be the target organs of TMUV. The level of viral loads was consistent with the severity of clinical signs and microscopic lesions in different tissues. The neutralizing antibody began to seroconvert at 8 dpi. Clinical signs, microscopic lesions, viral loads and serum neutralizing antibodies weren’t observed in other groups. In summary, TMUV can cause systemic infections and death in Kunming mice by i.c., which provides some experimental basis for further study of the significance of TMUV in public health.

## Introduction

Outbreaks of Tembusu virus (TMUV) infection in ducks have been reported since April 2010 in China, which resulted in greater economic losses to the poultry industry (Cao et al., 2011; Su et al., 2011; Yan et al., 2011). This disease was mainly characterized by a decline in egg production, follicular rupture and bleeding, and yolk peritonitis in laying ducks. Ducklings mainly demonstrated retarded growth, standing instability and paralysis, with 10–30% mortality rates (Tang et al., 2012; Yun et al., 2012). TMUV can infect Beijing duck, Shaoxing duck, golden duck, Cherry Valley, Jinyun duck, Campbell ducks and other species of ducks. Chickens, geese, sparrows and some birds were also infected and displayed clinical signs (Liu et al., 2012a; Li et al., 2013; Tang et al., 2015). So far, this disease has spread throughout the major duck-producing regions in China and caused a great threat to the duck industry. The inactivated vaccines and live attenuated vaccines against TMUV have been successfully developed and already used in clinical production, which provides a guarantee for better prevention and treatment of the disease (Li et al., 2014; Lin et al., 2015).

TMUV is a mosquito-borne *Flavivirus* of the *Ntaya* virus group which is classified into the *Flavivirus* genus, *Flaviviridae* family (Su et al., 2011). The *Flavivirus* genus includes more than 70 viruses, for example West Nile virus, Japanese encephalitis virus, Yellow fever virus, Dengue virus, etc, most of which can cause public health problems (Liu et al., 2013). There were approximately 50∼100 million people infected with dengue virus in more than 80 countries around the world annually and 10∼20% infections showed typical symptoms (Gubler, 2002, 2006). Japanese encephalitis virus can also cause 50,000∼175,000 cases of human infections annually and 20∼30% of cases were fatal and 30∼50% of survivors still exhibited severe complications even years later (Campbell et al., 2011). Other viruses such as Yellow fever virus, Saint Louis encephalitis virus, Murray Valley virus, and Ilheus virus also have a serious impact on public health (Fernandez-Garcia et al., 2009; de Barros et al., 2011).

It has aroused a widespread concern that whether TMUV as a mosquito-borne virus in the flavivirus genus can also infect mammals and cause disease. Only the ducks were infected when TMUV first appeared in China. Less than 1 year, the virus quickly spread to the geese, chickens and birds (Li et al., 2012; Liu et al., 2012b; Tang et al., 2013). It can be seen that the virus spread fast and had more susceptible animal species (Dai et al., 2015). It also suggests that more other species of animals may be infected with the virus, even the mammal. It was reported that Balb/c mice could be infected with the virus by intracerebral inoculation (Li et al., 2013). And TMUV could cause an antibody-dependent infection to Balb/c mice (Liu et al., 2013). On the basis of the previous study, we carried out some research works. In this study, we used the Kunming mice as our experimental animals because their biological characteristics are more similar to human and other mammals in the natural state (Chen et al., 2004). So Kunming mice are widely used to study on the microbial etiology, pathogenicity, pathogenesis, etc. in China. In this present study, we explored the pathogenicity of TMUV to Kunming mice using different ways of artificial inoculation.

## Materials and Methods

### Animal and Virus

Three-week-old female Kunming mice were purchased from Experimental Animal Center of Shandong Province (Jinan, China). The TMUV strain was isolated from a duck farm in Shandong Province in 2013. After three passages in the allantoic cavities of 9-day-old SPF duck embryos, the virus was used as the challenge virus for this study. The challenge virus was 10^4.8^ ELD_50_/0.2 ml (Median embryo lethal dose), calculated according to the Reed and Muench method (Reed and Muench, 1938), and named SDSG (Accession number: KJ740747.1). The animal experiment was approved by the Committee on the Ethics of Animal of Shandong (permit number: 20127620).

### Animal Experiments

Sixty 3-week-old female Kunming mice were separated into four groups. Three groups as the experimental groups (*n* = 15 mice per group) were inoculated with the challenge virus in 50 μL allantoic fluid (10^4.8^ ELD_50_/0.2 ml) respectively by the intracerebral (i.c.), subcutaneous (s.c.) and intranasal (i.n.) routes. One group as the control (*n* = 15 mice) was inoculated with 50 μL sterile phosphate-buffered saline (PBS). This challenge virus dose was determined by a preliminary animal test. Mice were monitored for changes in weight and typical clinical symptoms over 14 days. Serum samples of mice were collected and stored in -80°C until use. At 4, 8, 12 dpi, three mice in each group were euthanized and tissues (brain, spleen, liver, lung, kidneys, and intestine) were collected. One part of tissue samples was immediately fixed in 10% neutral buffered formalin solution for histological examinations and the other part of the tissue samples was stored at -80°C until use.

### RNA Extraction and Reverse Transcription

0.5 g of frozen tissues were homogenized and diluted in PBS for RNA extraction. Total RNA was obtained from the collected tissue samples with Trizol (TransGen, Beijing, China) according to the manufacturer’s instructions. Total RNA concentration of every sample was determined by measuring OD values at the wavelength of 260 and 280 nm by a spectrophotometer (Eppendorf, Germany). Complementary DNA (cDNA) of 1000 ng RNA was synthesized in 20 μL volume of reverse transcription system using the Prime Script 1st strand cDNA Synthesis Kit (TaKaRa, DaLian, China).

### Detection of Viral Loads in Different Tissues

SYBR Green I real-time PCR assay was used to detect the viral load in tissue samples. E gene as a target gene and mouse β-actin gene as a reference gene were detected in different tissues to analyze the viral RNA expression. Two pairs of primers were designed using the primer 5.0 software according to the gene sequences in GenBank (E gene, accession number KJ740747.1; β-actin, accession number NM_007393.4). The primers of E gene were as follows, E-F:5′-CGCTGAG ATGGAGGATTATGG-3′ and E-R:5′-ACTGATTGTTTGGTGGCGTG-3′. The primers of β-actin gene were as follows, β-F:5′-TGACAGGATGCAGAAGGAGA-3′ and β-R:5′- GCTGGAAGGTGGACAGTGAG -3′. The 25 μL PCR system contained 12.5 μL of 2 × TransStart SYBR Green qPCR SuperMix (TransGen, Beijing, China), 2 μL of cDNA template, 0.5 μL forward primer (10 μM), 0.5 μL reverse primer (10 μM), and 9.5 μLsterilized double-distilled water. The PCR thermal cycles were 95°C for 7 min, followed by 40 cycles of 94°C for 15 s and 59°C for 40 s, and collected fluorescence for 40 s at 59°C. Each sample was performed in triplicate.

### Histopathology and Immunohistochemistry Examinations

The different tissues were fixed in 10% neutral buffered formalin solution for at least 48 h and processed routinely for histopathology. After dehydration, fixed tissues were embedded in parafin wax and cut into 4-μm-thick sections. The sections were stained with hematoxylin and eosin (H&E) according to the staining procedure. Microscopic changes in different tissues were observed under the Microscope (Lycra, German). For immunohistochemistry examination, sections were deparaffinized through xylene and hydrated with ethanol. Sections were immersed in 3% hydrogen peroxide solution to inactive endogenous peroxidase and heated to expose antigen at 98∼100°C. Viral staining was performed with a mouse-derived monoclonal antibody for TMUV protein E (Chen et al., 2014) overnight at 4°C. After three washes in PBS, sections were incubated with a goat anti-mouse HRP-conjugated polyclonal serum (Beijing CoWin Biotech Co. Ltd., Beijing, China) for 1 h at 37°C. Diaminobenzidine was used as the substrate chromagen and sections were counterstained with hematoxylin. The sections were coverslipped with gum and observed under the microscope.

### Detection of Serum Neutralizing Antibodies Against TMUV

The serum neutralizing antibody titers were determined by Serum neutralization test (SNT). The complements in serum samples were inactivated before the test. Nine-day-old SPF chicken embryos were used for SNT as previously described (Li et al., 2012). Serum neutralizing antibody titers were expressed as the reciprocal of the highest dilution serum dilution that showed 50% SPF chicken embryos death. Serum antibody titers were determined using Reed and Muench method (Reed and Muench, 1938). Each sample was detected in triplicate.

### Statistical Analysis

Data were expressed as means** ±** standard deviation. The data of body weight and serum antibody titers were analyzed using two-tailed Student’s unpaired *t*-test. One-way analysis of variance with Tukey’s post-test was used to evaluate the viral load in different tissues using Graph Prism software (GraphPad Software Inc.). Statistical significance was set at *P* < 0.05.

## Results

### Clinical Signs and Weight Changes

**FIGURE 1:**
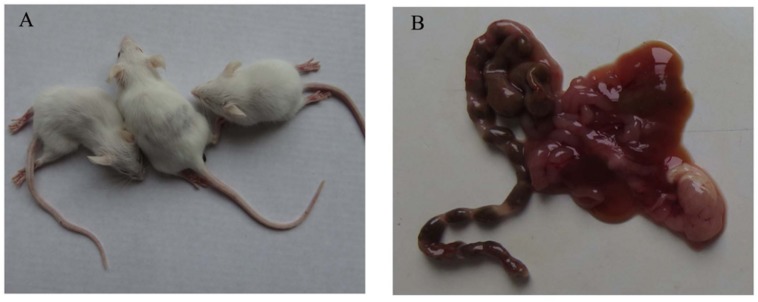
**Clinical signs and gross lesions of mice infected with TMUV by i.c.** **(A)** Three mice displayed paralysis of two hind legs, crawl at 8 dpi. **(B)** Liquefaction and hemorrhage of intestine were visible in the dead mice.

**FIGURE 2:**
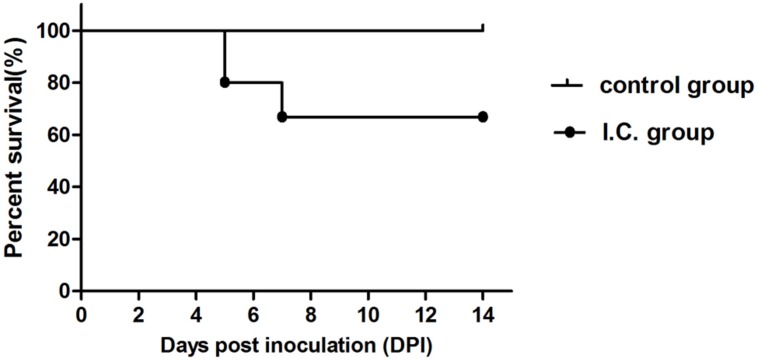
**The survival rate of Kunming mice infected with TMUV by i.c.** At 5 dpi, three mice died in the i.c. group. At 7 dpi, two mice died in the i.c. group.

**FIGURE 3:**
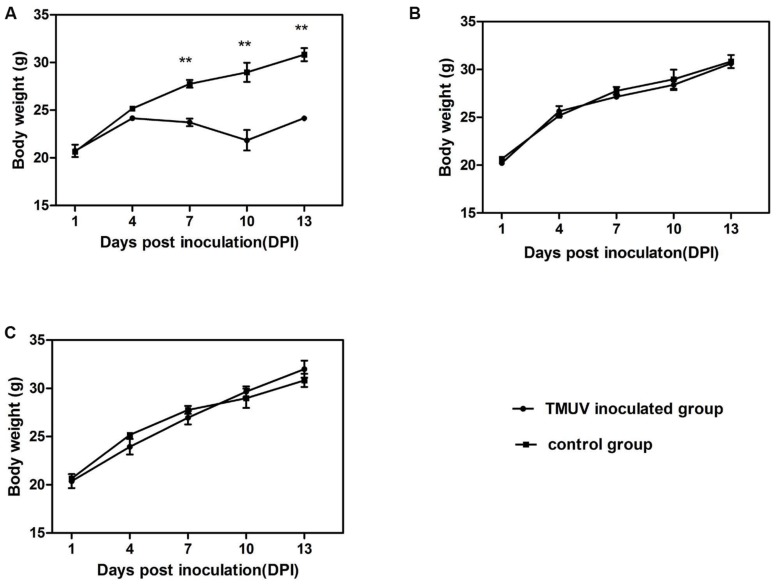
**Comparison of body weight changes for mice inoculated with TMUV by different routes.** The i.c. group mice **(A)**, the i.a. group mice **(B)**, the i.h. group mice **(C)** were inoculated with 50 μL × 10^4.8^ ELD_50_/0.2mL of the challenge virus. Bars denote means ± standard deviation (SD). The mean value was statistically significant, calculated by the two-tailed Student’s unpaired *t*-test (^∗∗^*P* < 0.01).

Clinical signs and body weight of four different groups were observed and recorded for 14 days. In the i.c. group, mice exhibited significant clinical symptoms, including depression, reluctant activities, anorexia, dried faces and yellow urine at 4 dpi. Two mice displayed paralysis of two hind legs, crawl at 4 dpi and three mice paralyzed at 8 dpi (**Figure 1A**). Five mice died throughout the experiment (**Figure 2**). Body weight gain started to decrease from 5 dpi compared with the control group and increase again till to 10 dpi. The mean body weight decreased to 5.42∼26.16% of that in the control group (**Figure 3A**). From 10 dpi, drinking and eating of the remaining mice began to recover gradually. In the s.c. and i.n. groups, mice didn’t show any clinical signs and the body weight gain was consistent with the control group during the 14 days (**Figures 3B,C**).

### Gross and Microscopic Lesions

In the i.c. group, splenomegaly and brain swelling were observed and no gross lesions were observed in other tissues. Dead mice demonstrated more severe gross lesions due to serious illness, such as liquefaction and hemorrhage of intestine (**Figure 1B**), splenic atrophy and meningeal edema. No obvious gross lesions were found in the s.c and i.n. groups.

**FIGURE 4:**
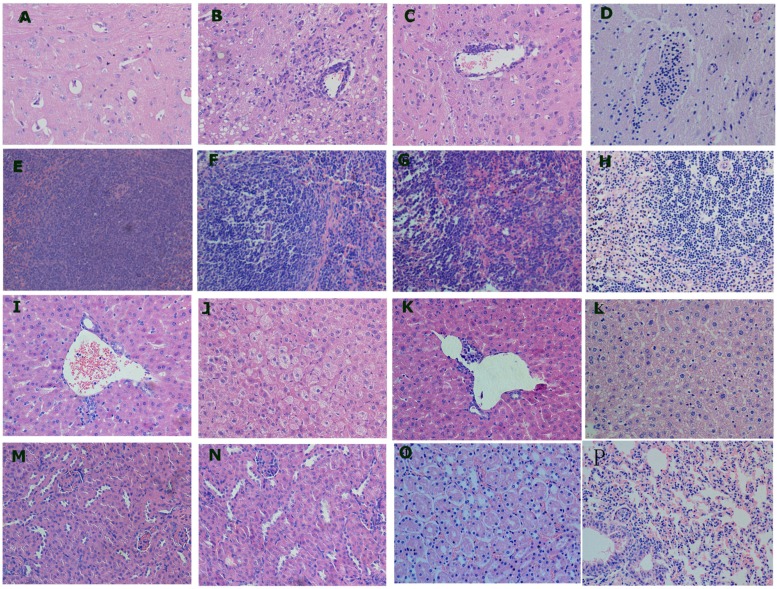
**Microscopic pathological changes of 3-week-old mice inoculated with TMUV by i.c.** **(A)** Brain, 4 dpi: microglia cell proliferation and capillary edema; **(B,C)** Brain, 8 and 12 dpi: severe lymphoid perivascular cuffing, perivascular lymphoid infiltration and fatty degeneration of nerve cells; **(D)** Brain, dead mice: severe microglial nodules and perivascular cuffing formation; **(E)** Spleen, 4 dpi: endothelial swelling; **(F,G)** Spleen, 8 and 12 dpi: varying degrees of lymphoid cell depletion; **(H)** Spleen, dead mice: severe lymphoid cell depletion; **(I,K)** Liver, 4 and 12 dpi: perivascular inflammatory cells infiltration; **(J)** Liver, 8 dpi: moderate necrosis of hepatocytes; **(L)** Liver, dead mice: multifocal necrosis of hepatocytes; **(M,N)** Kidney, 8 and 12 dpi: narrowing tubular lumen and epithelial cell shedding; **(O)** Kidney, dead mice: severe and extensive tubular epithelial cell shedding; **(P)** Lung, 8 dpi: interstitial pneumonia with inflammatory cells infiltration and capillary congestion of alveoli.

The main microscopic lesions were varying degrees of edema, congestion, fatty degeneration and lymphocyte infiltration in different tissues of the i.c. group mice. In the brain, mice demonstrated microglia cell proliferation and capillary edema at 4 dpi. Perivascular gap and pericellular gap were significantly widened (**Figure 4A**). At 8 dpi, more severe lesions were observed, such as severe lymphoid perivascular cuffing, perivascular lymphoid infiltration and fatty degeneration of nerve cells (**Figure 4B**). At 12 dpi, the severity of microscopic lesions decreased and lesions were similar to those at 8 dpi (**Figure 4C**). The lesions in the brain of dead mice were characterized by severe microglial nodules and perivascular cuffing formation (**Figure 4D**).

In the spleen, the i.c. group mice mainly demonstrated lymphoid cell depletion. At 4 dpi, endothelial swelling was frequently seen in the spleen (**Figure 4E**). At 8 dpi and 12 dpi, varying degrees of lymphoid cell depletion were the prominent features (**Figures 4F,G**). A severe lymphoid cell depletion was also observed in the splenic germinal center of dead mice (**Figure 4H**).

In the liver, the i.c. group mice displayed perivascular inflammatory cells infiltration at 4 dpi and 12 dpi (**Figures 4I,K**). At 8 dpi, moderate necrosis of hepatocytes was visible (**Figure 4J**). The multifocal necrosis of hepatocytes was frequently seen in the dead mice (**Figure 4L**). Narrowing tubular lumen and epithelial cell shedding were observed in the kidney at 8 and 12 dpi (**Figures 4M,N**). Tubular epithelial cell shedding was more severe and extensive in the dead mice (**Figure 4O**).

In the lung, mice mainly demonstrated interstitial pneumonia with inflammatory cells infiltration and capillary congestion of alveoli at 8 and 12 dpi (**Figure 4P**). The severe lesions were observed in the lung of dead mice. No obvious microscopic lesions were observed in the s.c and i.n. group mice.

### Immunohistochemistry Examinations

**FIGURE 5:**
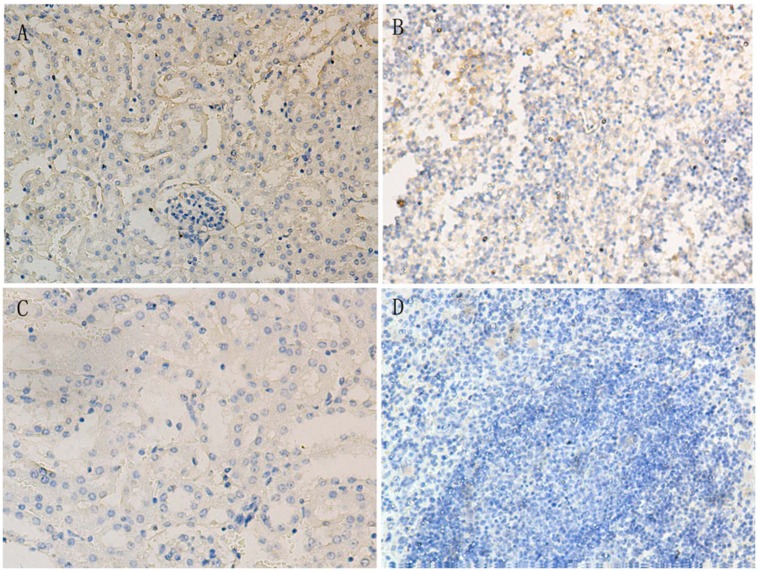
**Immunohistochemical examination of TMUV antigen in the kidney and spleen of Kunming mice at 8 dpi.** **(A)** kidney of mice infected intracerebrally; **(B)** spleen of mice infected intracerebrally; **(C)** kidney of the control mice; **(D)** spleen of the control mice.

In the i.c. group, immunohistochemical staining of kidney sections revealed that the positive viral signals were mainly distributed in renal tubular interstitium and renal tubular epithelial cells (**Figure 5A**). Positive signals were also observed in splenic cells (**Figure 5B**). The positive staining signals were distributed more widely in the mice infected intracerebrally at 8 dpi. No positive signals were found in the other group mice (**Figures 5C,D**).

### Viral Loads in Different Tissues

**FIGURE 6:**
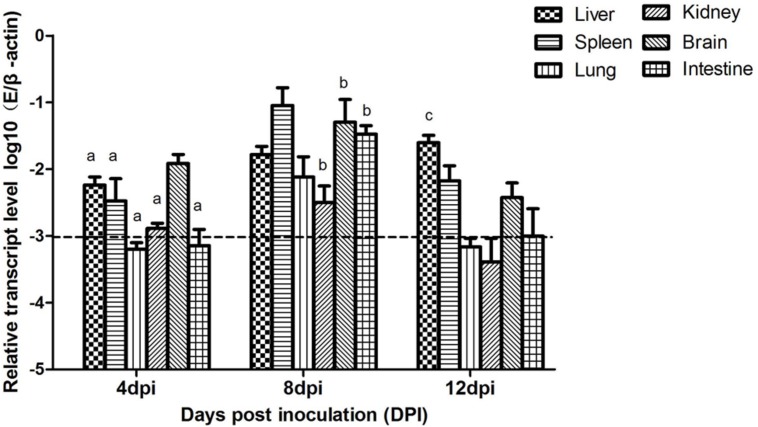
**Viral loads in tissues of mice on different days after inoculation by i.c.** The relative expression of viral RNA in tissues was determined by SYBR Green I real-time PCR assay (E gene as a target gene and mouse β-actin gene as a reference gene). Each sample was detected in triplicate. Error bars were expressed as standard deviation of the means (*n* = 3). Data are expressed as the mean log10 (E/β-actin) ± SD. Horizontal dotted line represents viral loads of the control group. Different lowercase letters over the bars denote statistically significant differences (*P* < 0.05) between different days after infection calculated by the one-way analysis of variance with Tukey’s post-test. (a) 4 dpi vs. 8 dpi; (b) 8 dpi vs. 12 dpi; (c) 4 dpi vs. 12 dpi.

In this study, viral loads in different tissues of the mice were respectively detected by the SYBR Green I relative real-time PCR assay in four groups at 4, 8, and 12 dpi. The level of viral RNA expression in different tissues of the i.c. group mice was shown in **Figure 6**. As can be seen from **Figure 6**, viral loads in the spleen, brain and liver were higher at 4 dpi and reached the peak at 8 dpi. Especially viral loads in the spleen, brain, and intestine were the highest compared with the other tissues. At 12 dpi, viral loads declined and were barely detectable in the kidney, lung and intestine. But viral RNA in the spleen, brain and liver always remained at a high level and maintained a long time. Viral loads in different tissues from the s.c., i.n. and control group mice weren’t detected.

### Detection of the Serum Neutralizing Antibody

**FIGURE 7:**
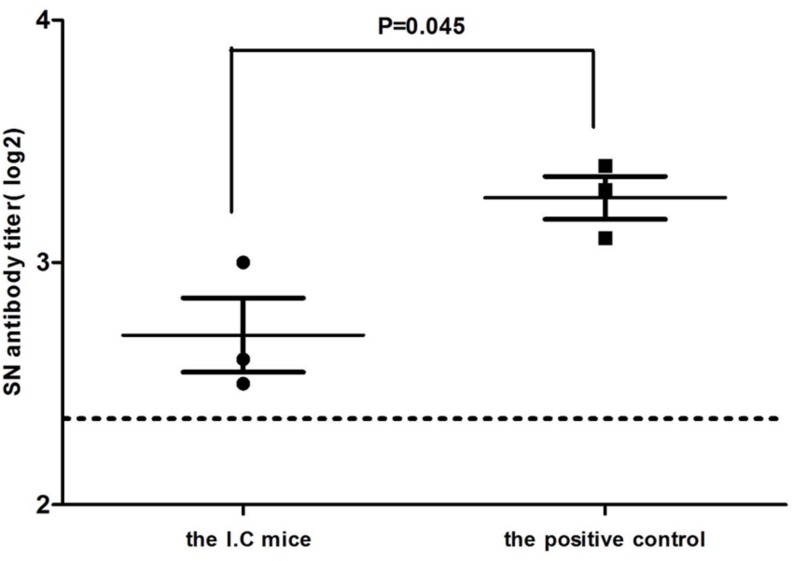
**Serum neutralizing antibody titers in the i.c. group were detected at 14 dpi.** Each sample was detected in triplicate. Antibody titers are expressed as the reciprocal of the log2 of the highest serum dilution that showed 50% SPF chicken embryos death. The dotted line represents negative antibody titers. Data are expressed as mean ± SD (*n* = 3). Significant differences were calculated by the two-tailed Student’ s unpaired *t*-test.

As indicated in **Figure 7**, Kunming mice inoculated with TMUV intracerebrally developed positive antibody titers at 8 dpi (SN antibody titer > 5). From 8 dpi, the neutralizing antibody titers were continuously ascending, but the level was low. No mice in the s.c and i.n. groups showed positive neutralizing antibody titers to TMUV (SN antibody titer <5).

## Discussion

Duck TMUV has been an emerging flavivirus that mainly infected waterfowls in China in recent years. Flaviviruses are zoonotic pathogens which have a higher pathogenicity on central nervous system (CNS). For example, Japanese encephalitis virus can cause a variety of neurological symptoms including mental status changes, focal neurologic deficits and movement disorders (Kalita and Misra, 2000; Solomon and Vaughn, 2002; Al-Mujadi et al., 2006; Kumar et al., 2006). The other flavivirus such as Dengue virus (Gupta et al., 2012) and Yellow fever virus (Kimura et al., 2010) also have the features of causing viral encephalitis in mouse infection models. For this novel pathogen TMUV, little information is available about its pathogenicity in mammal. In our study, the research was focused on the pathological changes and viral loads in different tissues from Kunming mice via different inoculation routes with a non-adapted Duck TMUV to evaluate the pathogenicity in Kunming mice.

It is reported that mice infected with flavivirus intracerebrally often show moderate to severe signs, such as paralysis and even death (Shresta et al., 2006; Yauch and Shresta, 2008). In this study, 3-week-old Kunming mice infected TMUV intracerebrally exhibited a decline of body weight, hindlimb paralysis and death. Histopathological changes in the brain were mainly characterized by varying degrees of viral encephalitis which was the most serious at 8 and 12 dpi. The experimental results indicate that Duck TMUV can invade the brain tissue and result in significant neurological symptoms and pathological changes in Kunming mice by i.c. route. This further illustrates that TMUV possesses the ability of perineural invasion like other flavivirus.

Besides the brain, TMUV can also cause more severe pathological changes in the other tissues. The liver, spleen, lung, kidney, and intestine of Kunming mice in i.c. group also showed typical pathological changes. Microscopic lesions in the liver were characterized by lymphocyte infiltration, hepatocyte edema and mild hepatic steatosis. Pathological changes in the spleen mainly included varying degrees of lymphocyte depletion. Pathological changes in the kidney were very slight. Only renal tubular exhibited mild swelling, narrowing tubular lumen and epithelial cell shedding. These features were also observed in the cases caused by other flavivirus. For example, Japanese encephalitis virus can invade the liver and spleen and cause viremia through the blood circulation (Wang and Liang, 2015). Dengue virus can cause varying degrees of disease from mild asymptomatic illness to severe fatal diseases (Gupta et al., 2012). In previous studies on pathogenicity of TMUV in ducks, these lesions were also observed in different tissues (Sun et al., 2014).

It is reported that pathological changes of the brain and spleen were worst in mice infected with Japanese encephalitis by i.c. at 8∼10 dpi (Grossberg and Scherer, 1966). In our study, pathological changes in the brain, spleen, and liver were the most serious at 8 and 12 dpi, which was consistent with the level of viral load in these tissues. At 8 dpi, the viral RNA in each tissue reached the highest level and the viral load in brain and spleen maintained a longer time. These data indicated that the brain, spleen and liver of Kunming mice may be the target organs of TMUV. The results were in agreement with other studies (Su et al., 2011; Liu et al., 2013). Previous reports indicated that TMUV could infect Balb/c mice but the virus could be detected only from the brain, liver, spleen and kidney (Li et al., 2013). In this study, the lung and intestine of Kunming mice infected intracerebrally demonstrated significant microscopic lesions and viral loads. It is further suggested that Kunming mice may be more susceptible to the virus and Kunming mouse will be a better animal model which is used to study the pathogenicity of TMUV on mammals.

Previous studies showed that serum neutralizing antibody titers were lower or even absent in the ducklings inoculated with TMUV at 5 days of age (Sun et al., 2014). In the present study, neutralizing antibodies titers to TMUV in Kunming mice were also low and developed late. The low antibodies may be related to the inoculation routes of virus. Mice inoculated with Japanese encephalitis virus intracerebrally developed much lower neutralizing antibodies than those inoculated intramuscularly (Sun et al., 2012). Another reason could be due to immaturity of the immune system of young mice or the severe immunosuppression caused by lymphoid cell depletion in the spleen (Eldadah et al., 1967; Jones and Kibenge, 1984).

## Conclusion

Kunming mice can be infected with an untamed TMUV strain to result in disease or even death by a specific route. The virus was rapid reproductive in the tissues of mice and especially maintained for a long time in the brain, liver, spleen, kidney, lung, and intestine. TMUV showed more severe pathogenicity in Kunming mice than Balb/c mice (Li et al., 2013) which indicated that Kunming mice were also used as the model animals to further study the pathogenesis or immune responses of TMUV. Moreover, the price of Kunming mice was far lower than that of Balb/c mice which can reduce the experimental outlay greatly. So, this study provides some experimental data for further exploration of the pathogenic mechanisms of TMUV in mammals.

## Author Contributions

Each author has taken part in the study.

## Conflict of Interest Statement

The authors declare that the research was conducted in the absence of any commercial or financial relationships that could be construed as a potential conflict of interest.
